# Psychological and financial impact of the COVID-19 pandemic during the first stages of the pandemic: Brazilian orthodontists´ perspective

**DOI:** 10.1590/2177-6709.27.6.e2221219.oar

**Published:** 2023-03-27

**Authors:** Ludmila Mangialardo LIMA, Aron ALIAGA-DEL CASTILLO, Camila MASSARO, Caroline Martins GAMBARDELA, Deborah Brindeiro de Araújo BRITO, Felicia MIRANDA, Lorena VILANOVA, Paula COTRIN, Raquel Silva POLETTO, Wilana MOURA, Arnaldo PINZAN, Guilherme JANSON, Fabiola Alvarez AVILA

**Affiliations:** 1 Universidade de São Paulo, Faculdade de Odontologia de Bauru, Departamento de Ortodontia (Bauru/SP, Brazil).; 2 University of Michigan, School of Dentistry, Department of Orthodontics and Pediatric Dentistry (Ann Arbor, USA).

**Keywords:** COVID-19, Psychology, Orthodontics

## Abstract

**Introduction:**

Brazil faced a catastrophic situation in the coronavirus pandemic. Due to the high risk of contamination and spread of COVID-19, dentist have been attending only urgency and emergency services in Brazil at the beginning of the pandemic.

**Objective:**

This research aimed to evaluate the psychological and financial impacts caused by the coronavirus pandemic in Brazilian orthodontists.

**Methods:**

This population-based cross-sectional study collected demographic data and mental health measurements from 404 orthodontists. Depression, anxiety, insomnia, and distress were evaluated through Brazilian versions of the 9-item Patient Health Questionnaire (9-PHQ), the 7-item Generalized Anxiety Disorder scale and Mini-Tracking (GAD), the 7-item Insomnia Severity Index (ISI), and the 22-item Impact of Event Scale-Revised (IES-R), respectively. The demographic data of the sample was described using descriptive statistics. The data was analyzed according to sex, professional status, and economic income. Comparisons were performed using Chi-square tests, Mann-Whitney U tests, and Kruskal-Wallis followed by *post-hoc* tests.

**Results:**

Females, graduate students, and lower incomes subgroups showed higher levels of depression, anxiety, insomnia, and distress. Most orthodontists showed moderate to extreme financial and professional concerns during the pandemic.

**Conclusion:**

The coronavirus pandemic negatively affected the psychological health and increased the financial concerns of the Brazilian orthodontists, mainly female, graduate students, and with income below 10k participants.

## RESUMO

**Introdução:**

O Brasil enfrentou uma situação catastrófica durante a pandemia do coronavírus. Devido ao alto risco de contaminação e disseminação do vírus da COVID-19, os cirurgiões-dentistas passaram a realizar apenas atendimentos de urgência ou emergência no início da pandemia.

**Objetivo:**

O presente estudo teve como objetivo avaliar o impacto financeiro e psicológico causado pela pandemia do coronavírus nos ortodontistas brasileiros.

**Métodos:**

Este estudo transversal de base populacional coletou os dados demográficos e o impacto da pandemia em 404 ortodontistas. Depressão, ansiedade, insônia e angústia foram avaliadas, respectivamente, por meio da versão em português do Questionário de Saúde do Paciente (PHQ-9), do módulo de transtorno de ansiedade generalizada (GAD) do Mini-Tracking (GAD/Mini-Tracking), do Índice de Severidade de Insônia (ISI) e o do Impact of Events Scale-Revised (IES-R). As características demográficas da amostra foram apresentadas por meio de estatística descritiva. Os dados foram analisados de acordo com o sexo, situação profissional e renda econômica. As comparações foram realizadas utilizando os testes de Qui-quadrado, Mann-Whitney U e Kruskal-Wallis, seguidos de testes *post-hoc* (*p*<0,05).

**Resultados:**

Mulheres, estudantes de pós-graduação e profissionais com menores rendas apresentaram níveis mais altos de depressão, ansiedade, insônia e angústia. A maioria dos ortodontistas mostrou preocupação financeira e profissional moderada a extrema durante a pandemia.

**Conclusão:**

A pandemia do coronavírus afetou negativamente a saúde psicológica dos ortodontistas brasileiros e aumentou as preocupações financeiras desses profissionais. As mulheres, os estudantes de pós-graduação e os participantes com renda mensal menor que R$10 mil foram os grupos mais afetados.

## INTRODUCTION

In March 2020, the World Health Organization (WHO) declared the coronavirus pandemic, due to the significant increase in the number of reported cases and the global virus spread.^1^ In the middle of 2020, Brazil was the epicenter of the coronavirus outbreak in Latin America, presenting the second-highest number of cases and deaths in the world. Until the end of July 2020, more than 2 million cases were confirmed, and 90 thousand deaths were reported in Brazil.^2^

In dental practice, both patients and professionals are exposed to a high risk of COVID-19 infections. The frequent exposure to saliva and blood, the proximity between patient and professional, and the aerosol spread increase the contamination risks.^3^ ^,^ ^4^

For this reason, dentists attended only urgency and emergency services during the coronavirus pandemic in Brazil, at the beginning of the pandemic. Elective treatments such as Orthodontics have been postponed until the situation becomes controlled.^5^ ^,^ ^6^

Concerns regarding contamination of dentists due to the transmission of the coronavirus through saliva were previously reported, and safety measures were recommended.^4^ ^,^ ^7^ ^-^ ^9^ In this context, the coronavirus pandemic may cause physical and mental effects on health workers. The pandemic situation negatively affects psychological health and financial status.^10^ ^,^ ^11^ Symptoms as post-traumatic stress, depression, anxiety, insomnia, and emotional exhaustion have been reported.^10^ ^-^ ^13^

Therefore, this study aimed to evaluate the psychological, financial, and professional impacts during the first stages of the coronavirus pandemic in Brazilian orthodontists.

## MATERIAL AND METHODS

This population-based cross-sectional study was approved by the Ethics Committee on Human Research of University of São Paulo (protocol number N. 4.023.156 CAAE 30984620.6.0000.5417), and all participants agreed to participate in the survey. Their identities were kept confidential.

The sample size was calculated considering 80% of test power, a significance level of 5%, a design effect of 1, and 50% frequency of psychological symptoms, based on a previous study of the COVID-19 outbreak.^7^ According to the Brazilian Federal Council of Dentistry, the population of orthodontists comprised 27940 subjects.^14^ Therefore at least 379 completed questionnaires were necessary. 

The data was obtained in early May 2020 through the Google Forms platform (Google Inc, Mountain View, CA, USA). The questionnaire was sent by e-mail or WhatsApp Messenger (Facebook Inc, Mountain View, CA, USA) to Brazilian orthodontists and postgraduate students. The link was available for 10 days. Participants could refuse or withdraw to participate at any time, leaving the website, without any penalty or loss.

The survey was composed by questionnaires with multiple-choice answers to evaluate the demographic data, mental health, and impact in finances and professional activities.

The symptoms of depression, anxiety, insomnia and distress for all participants were assessed through previously validated questionnaires: the Patient Health Questionnaire (PHQ-9),^15^ ^-^ ^17^ the generalized anxiety disorder (GAD) module of the Mini-Tracking (GAD/Mini-Tracking),^18^ ^-^ ^20^ Insomnia Severity Index (ISI),^21^ Impact of Events Scale-Revised (IES-R),^22^ ^,^ ^23^ respectively.

PHQ values ranged from 0 to 27, and were classified as (0-4) normal, (5-9) mild, (10-14) moderate, (15-27) severe; GAD/Mini-tracking values ranged from 0 to 32 and were classified as (0-8) normal, (9-16) mild, (17-24) moderate and (25-32) severe; ISI scores ranged from 0 to 28, and were classified as (0-7) absence, (8-14) subthreshold, (15-27) moderate and (22-28) severe; IES-R scores ranged from 0 to 88, and were classified as (0-8) normal, (9-25) mild, (26-43) moderate and (44-88) severe. These classifiers were adapted from previous studies.^7^ ^,^ ^15^ ^-^ ^23^

**Figure 1:**
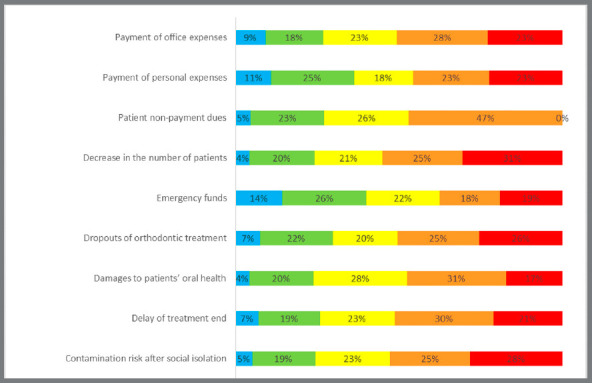
Level of concern in Orthodontics about financial and professional activities during the coronavirus pandemic.

All participants also answered 10 additional questions regarding the impact of coronavirus pandemic in financial and professional concerns. The economic incomes were evaluated in the local currency (Fig 1).

### STATISTICAL ANALYSIS

The demographic data of the sample was described using descriptive statistics. The data was analyzed according to sex, professional status, and economic income. Comparisons were performed using Chi-square tests, Mann-Whitney U tests, and Kruskal-Wallis followed by *post-hoc* tests (*p*<0.05). Statistical analyses were performed with SPSS statistical software package (version 21.0; SPSS, Chicago, IL).

## RESULTS

The sample was composed by 404 orthodontists (259 females; 145 males). The demographic characteristics of the sample are described in Table 1. 

**Table 1:** Demographic and professional characteristics of responders.

	n (%)						
	TOTAL	PROFESSIONAL STATUS			ECONOMIC INCOME		
		Professor	Graduate student	Clinician	<5K	5-10k	> 10k
Overall	404 (100.0)	152 (37.6)	115 (28.5)	137 (33.9)	97 (24.0)	134 (33.2)	173 (42.8)
Sex							
Female	259 (64.1)	77 (50.7)	84 (73.0)	98 (71.5)	79 (81.4)	100 (74.6)	80 (46.2)
Male	145 (35.9)	75 (49.3)	31 (27.0)	39 (28.5)	18 (18.6)	34 (25.4)	93 (53.8)

The severity of depression, anxiety, insomnia, and distress is reported in Table 2. Most of the sample presented mild to severe symptoms of depression (62.4%), anxiety (62.6%), insomnia (50.8%), and distress (82.4%) (Table 2).

**Table 2:** Descriptive statistics of the severity categories of depression, anxiety, insomnia, and distress in Brazilian orthodontists during COVID-19 pandemic.

Questionnaire	Total n (%)	Sex - n (%)			Professional status - n (%)				Economic income - n (%)	
		Female	Male	Professor	Graduate	Clinician	<5k	5-10k	>10k
PHQ-9 - depression symptoms									
Normal (0-4)	128 (31.7)	51 (19.7)	77 (53.1)	69 (45.4)	19 (16.5)	40 (29.2)	11 (11.3)	39 (29.1)	78 (45.1)
Mild (5-9)	121 (30.0)	83 (32.0)	38 (26.2)	41 (27.0)	36 (31.3)	44 (32.1)	30 (30.9)	35 (26.1)	56 (32.4)
Moderate (10-14)	80 (19.8)	65 (25.1)	15 (10.3)	23 (15.1)	26 (22.6)	31 (22.6)	24 (24.7)	31 (23.1)	25 (14.5)
Severe (15-27)	75 (18.6)	60 (23.2)	15 (10.3)	19 (12.5)	34 (29.6)	22 (16.1)	32 (33.0)	29 (21.6)	14 (8.1)
GAD module (Mini-Tracking) - anxiety symptoms									
Normal (0-8)	151 (37.4)	70 (27.0)	81 (55.9)	75 (49.3)	25 (21.7)	51 (37.2)	20 (20.6)	45 (33.6)	86 (49.7)
Mild (9-16)	131 (32.4)	94 (36.3)	37 (25.5)	38 (25.0)	45 (39.1)	48 (35.0)	36 (37.1)	43 (32.1)	52 (30.1)
Moderate (17-24)	95 (23.5)	74 (28.6)	21 (14.5)	32 (21.1)	33 (28.7)	30 (21.9)	31 (32.0)	33 (24.6)	31 (17.9)
Severe (25-32)	27 (6.7)	21 (8.1)	6 (4.1)	7 (4.6)	12 (10.4)	8 (5.8)	10 (10.3)	13 (9.7)	4 (2.3)
ISI - insomnia symptoms									
Absence (0-7)	199 (49.3)	108 (41.7)	91 (62.8)	84 (55.3)	42 (36.5)	73 (53.3)	37 (38.2)	60 (44.8)	102 (59.0)
Subthreshold (8-14)	134 (33.2)	96 (37.1)	38 (26.2)	44 (28.9)	47 (40.9)	43 (31.4)	36 (37.1)	50 (37.3)	48 (27.7)
Moderate (15-21)	65 (16.1)	51 (19.7)	14 (9.7)	20 (13.2)	26 (22.6)	19 (13.9)	22 (22.7)	23 (17.2)	20 (11.6)
Severe (22-28)	6 (1.5)	4 (1.5)	2 (1.4)	4 (2.6)	0 (0.0)	2 (1.5)	2 (2.1)	1 (0.7)	3 (1.7)
IES-R - distress symptoms									
Normal (0-8)	71 (17.6)	35 (13.5)	36 (24.8)	41 (27.0)	11 (9.6)	19 (13.9)	12 (12.4)	22 (16.4)	37 (21.4)
Mild (9-25)	162 (40.1)	94 (36.3)	68 (46.9)	62 (40.8)	36 (31.3)	64 (46.7)	34 (35.1)	51 (38.1)	77 (44.5)
Moderate (26-43)	112 (27.7)	86 (33.2)	26 (17.9)	31 (20.4)	47 (40.9)	34 (24.8)	34 (35.1)	37 (27.6)	41 (23.7)
Severe (44-88)	59 (14.6)	44 (17.0)	15 (10.3)	18 (11.8)	21 (18.3)	20 (14.6)	17 (17.5)	24 (17.9)	18 (10.4)

PHQ-9 = 9-item Patient Health Questionnaire; GAD = Generalized Anxiety Disorder; ISI = Insomnia Severity Index; IES-R = Impact of Event Scale-Revised.

Statistically significant differences were observed in all compared categories (Table 3). Greater median scores were observed for females, graduate students, and professionals with lowest incomes (<10k).

**Table 3:** Total scores of depression, anxiety, insomnia, and distress in orthodontists, and comparison between sex, professional status, and economic income range.

Questionnaire	TOTAL SCORE Median (IQR)	SEX Median (IQR)			PROFESSIONAL STATUS Median (IQR)				ECONOMIC INCOME Median (IQR)			
		Female	Male	*p* value	Professor	Graduate	Clinician	*p* value	<5k	5-10k	>10k	*p* value
PHQ-9 (0-27)	8 (4.0-13.0)	9 (5.0-14.0)	4 (2.0-8.0)	< 0.001*	5.5^A^ (2.0-10.0)	10.0^B^ (6.0-15.0)	7.0^A^ (4.0-12.5)	< 0.001*	10.0^A^ (7.0-16.0)	8.0^A^ (4.0-14.0)	5.0^B^ (2.0-9.0)	< 0.001*
GAD module (Mini-Tracking) (0-32), anxiety symptoms	11 (6.0-18.0)	14 (8.0-20.0)	7 (4.0-13.0)	< 0.001*	9.0^A^ (4.7-17.0)	14.0^B^ (9.0-20.0)	11.0^A^ (6.0-17.0)	< 0.001*	15.0^A^ (9.0-20.0)	12.5^A^ (6.0-19.0)	9.0^B^ (4.0-15.0)	< 0.001*
ISI (0-28), insomnia symptoms	8 (3.0-12.3)	9 (3.5-13.5)	5 (2.0-11.0)	< 0.001*	6.0^A^ (2.0-12.0)	10.0^B^ (5.0-14.0)	7.0^A^ (3.0-12.0)	< 0.001*	10.0^A^ (4.0-14.0)	9.0^A^ (3.2-12.8)	6.0^B^ (2.0-11.0)	< 0.001*
IES-R (0-88), distress symptoms	22.5 (12.0-36.0)	26 (14.0-39.0)	17 (9.0-27.0)	< 0.001*	18.5^A^ (8.0-29.0)	30.0^B^ (17.0-39.5)	21.0^A^ (12.0-34.0)	< 0.001*	26.0^A^ (17.0-41.0)	23.0^A^ (12.0-38.0)	18.0^B^ (10.0-29.0)	< 0.001*

* Statistically significant for *p* < 0.05. Different capital letters indicate statistically significant differences. PHQ-9 = 9-item Patient Health Questionnaire; GAD = Generalized Anxiety Disorder; ISI = Insomnia Severity Index; IES-R = Impact of Event Scale-Revised. IQR = interquartile range.

Distinct levels of concern were observed among orthodontists regarding the impact of the coronavirus pandemic in the financial and professional activities (Fig 1). Females demonstrated greater concern about payment of office expenses, delay of treatment end, contamination risks, and emergency appointments (Table 4). Professors demonstrated lower concern about patient’s dropouts and delays of treatment end, compared to graduate students and clinicians (Table 4). 

**Table 4:** Perception of orthodontics about income and appointments (Mann-Whitney and Kruskal-Wallis tests).

Questionnaire	TOTAL SCORE Median (IQR)	SEX Median (IQR)			ROFESSIONAL STATUS Median (IQR)				ECONOMIC INCOME Median (IQR)			
		Female	Male	***p* value**	rofessor	ostgraduate	Clinician	***p* value**	<5k	5-10k	>10k	***p* value**
ayment of office expenses (0-4)	3 (1-3)	3 (2-3)	2 (1-3)	0.032*	2 (1-3)	3 (2-4)	3 (2-3)	0.131	3 (2-3)	3 (2-4)	2 (1-3)	0.139
ayment of personal expenses (0-4)	2 (1-3)	2 (1-3)	2 (1-3)	0.131	2 (1-3)	2 (1-4)	2 (1-3)	0.077	2 (1-4)	2 (1-3)	2 (1-3)	0.42
atient non-payment dues (0-4)	2 (1-3)	3 (2-4)	3 (2-4)	0.32	2 (1-3)	3 (2-3)	2 (2-3)	0.219	3 (1-3)	2 (1-3)	2 (2-3)	0.917
Decrease in the number of patients (0-4)	3 (2-4)	3 (2-4)	3 (2-4)	0.23	2.0^A^ (1-3)	3.0^B^ (2-4)	3.0^B^ (2-4)	0.000*	3 (2-4)	3 (1-4)	2 (2-4)	0.061
Emergency funds (0-4)	2 (1-3)	2 (1-3)	2 (1-3)	0.164	2 (1-3)	2 (1-3)	2 (1-3)	0.499	2 (1-3)	2 (1-3)	2 (1-3)	0.302
Dropouts of orthodontic treatment (0-4)	3 (1-4)	3 (1-4)	2 (1-3)	0.061	2.0^A^ (1-3)	3.0^B^ (2-4)	3.0^B^ (1-4)	0.000*	3 (1-4)	2 (1-4)	2 (1-3)	0.117
Damages to patients’ oral health (0-4)	2 (2-3)	2 (2-3)	2 (1-3)	0.403	2 (2-3)	2 (2-3)	2 (1-3)	0.508	3 (2-3)	3 (1-3)	2 (2-3)	0.497
Delay of treatment end (0-4)	3 (1-3)	3 (2-3)	2 (1-3)	0.010*	2.0^A^ (1-3)	3.0^B^ (2-4)	3.0^AB^ (1-3)	0.006*	3.0^A^ (2-4)	3.0^AB^ (1-3)	2.0^B^ (1-3)	0.005*
Contamination risk after social isolation (0-4)	3 (2-4)	3 (2-4)	2 (1-3)	0.001*	3 (1-4)	3 (2-4)	2 (1-4)	0.092	3.0^A^ (2-4)	3.0^A^ (2-4)	2.0^B^ (1-3)	0.006*
Emergency appointments during the isolation period (0-4)	2 (1-3)	3 (1-4)	2 (1-3)	0.000*	2 (1-3)	2 (1-4)	2 (1-3)	0.222	3.0^A^ (1-4)	3.0^A^ (1-4)	2.0^B^ (1-3)	0.008*

* Statistically significant for *p* < 0.05.

Different capital letters indicate statistically significant differences.

Professionals with highest incomes (>10k) were less concerned regarding the delay of treatment end, contamination risks, and emergency appointments (Table 4). 

## DISCUSSION

To the best of our knowledge, this is one of the few studies that evaluated the psychological and financial impacts of the coronavirus pandemic in orthodontics. Moreover, it is essential to highlight that the results reported here are relevant to the time the study was conducted, that is, in the early stages of the pandemic in Brazil. Several studies conducted at the same time worldwide showed similar results. It could be stated that Orthodontics situation was similar all over the world. Most of the respondents reported perceived economic, psychosocial, and social impacts due to the pandemic.^24^ ^-^ ^27^

An electronic questionnaire was applied in a Brazilian orthodontic population and 404 responses were received. Female orthodontists comprised the majority of the sample (64.1%). This was expected since most of orthodontists in Brazil are female.^14^ Additionally, previous studies showed a greater female response prevalence in studies involving questionnaires with health professionals.^7^ ^,^ ^28^ ^-^ ^32^ Overall, most of the participants were professors or graduate students, suggesting that academic professionals were most predisposed to answer the questionnaire.

Outbreaks of infectious diseases cause a high psychological impact on health professionals.^28^ In the present study, the participants reported mild to severe symptoms of depression (68.4%), anxiety (62.2%) insomnia (50.7%), and distress (82.4%), which suggests a negative impact of COVID-19 pandemic in the mental health of orthodontists (Table 2). A similar study conducted in the USA during the same stages of the pandemic showed that dentists reported symptoms of depression and anxiety as well.^33^ Negative psychologic impact in healthcare workers have been previously reported during coronavirus pandemic.^7^ ^,^ ^11^ ^,^ ^29^ ^,^ ^32^ Also, anxiety and depression are very associated with sleep disturbances.^34^ ^,^ ^35^ Compared with other occupational groups, healthcare workers reported the highest rate of poor sleep quality.^36^ Therefore, the concern with mental health is directly related to physical health, and both need attention by the occupational health policies, during the pandemic COVID-19.^7^ ^,^ ^31^

Females were statistically significant more affected than males regarding all psychological symptoms reported in this study (Tables 2 and 3), which agrees with Gibson et al.^37^ They stated that female sex is one of the factors that could predict mental health inequalities during the COVID-19 pandemic. This was expected; since females usually report higher anxiety scores than males.^38^ ^,^ ^39^ During the coronavirus pandemic, previous studies showed that adult females also reported higher anxiety scores.^31^ ^,^ ^40^ In addition, moderate to severe symptoms of depression, anxiety, insomnia, and distress was found for female physicians and nurses.^7^ ^,^ ^41^ On the other hand, a similar study conducted in the same period in Turkey showed different results.^24^ The authors reported that only 16.7% of the orthodontists had anxiety symptoms, and there was no statistically significant difference when the prevalence of these symptoms was stratified by sex and age. It could be speculated that although both studies were conducted simultaneously, the countries were in different phases of the pandemic. One must also consider the geographic and cultural differences between the two countries. 

Graduate participants were significantly more affected than clinicians and professors regarding all psychological issues (Tables 2 and 3). Similarly, graduate students experienced a negative psychological impact of the coronavirus outbreak in China.^31^ The results of this study are in accordance with previous reports showing that younger people demonstrated a significantly higher prevalence of anxiety and depressive symptoms than older ones.^36^ ^,^ ^42^ ^,^ ^43^ So, it could be speculated that graduated students were more affected than clinicians and professors because they are usually younger.

The present research showed significantly greater psychological impact in professionals with income below 10k (Tables 2 and 3). In Brazil, the postponing of elective dental procedures caused a substantial reduction of orthodontics activities. According to Cotrin et al.^44^ the number of jobs reduced for all healthcare workers in Brazil during the first stages of the pandemic, but this reduction was significantly greater for dentists. It has been reported that people with financial stress are more vulnerable to mental health issues.^43^ A previous study with dental practitioners showed that the negative economic impact of the office closure was subsequently associated with concerns about professional future, anxiety, and fear.^11^ Additionally, lower incomes were previously associated with higher levels of distress.^43^ ^,^ ^45^ ^,^ ^46^

Most orthodontists presented moderate to extreme concerns regarding financial issues, including dropouts of orthodontic treatment and patient’s non-payment dues. Interestingly, they were extremely more concerned with patients’ dropouts than having a financial emergency personal fund (Fig 1). In addition, orthodontists also showed concern about contamination risks after social isolation (Fig 1). Coronavirus outbreak has affected all sectors in the economy all over the world, and it was not different in Brazil, which already presented a fragile economic situation before the pandemic. The New York Times recently identified dentists as in the highest risk of contamination at that time.^47^ Due to these facts, the postponing of the elective dental procedures was strongly recommended, resulting in severe monetary implications for dental practitioners worldwide.^48^ In addition, different from other countries, no governmental assistance was provided to Brazilian clinicians.^48^ ^,^ ^49^ Then, orthodontists can be expected to be dramatically concerned about the financial impact that the pandemic will cause during the restart of their dental practices.^50^

Regarding differences between sexes, the only financial issue that the female orthodontists were significantly more concerned than males was regarding the payment of office expenses (Table 4). This specific concern agrees with Ferneini,^51^ who evaluated the financial impact of COVID-19 in the dental practice. The author stated that the pandemic brought overhead costs because it required the orthodontic team to have a better and safer working environment for the patients, staff, and orthodontists. This will potentially increase orthodontists’ business overhead and reduce the profit margin even further.

When evaluating the impact of the coronavirus pandemic on the orthodontists’ professional lives, the females showed greater concern regarding the delay of treatment end, contaminations risks and emergency appointments than males (Table 4). These findings reinforce the greater emotional impact of the pandemic in female orthodontists (Table 3). These sex differences on risk and resilience to stress are complex and varies according to characteristics of the stressful factor, such as type, timing, and duration, as well as changes in brain structure and function.^39^ Moreover, health care providers are particularly vulnerable to emotional distress in the current pandemic, due to the exposure risks, and the additional concern about infecting their family and friends.^7^ ^,^ ^52^

Among the three evaluated groups, professors showed the lowest level of concern regarding patients’ dropouts, decreasing number of patients and delay of treatment end (Table 4). The delay of treatment end was also a recurring concern among patients during the pandemic.^53^ ^,^ ^54^ It could be thought that graduate students and clinicians have higher levels of patient-related concerns due to the nature of their clinical routine. In the other hand, professors usually have a greater workload dedicated to teaching and research.

Finally, orthodontists with higher incomes showed a lower level of concerns regarding the delay of treatment end, contamination risks and emergency appointments during the isolation period (Table 4). It is not surprising that financial security influences the behavior of orthodontists in other areas of their lives. Higher income has been reported as a beneficial factor for psychological wellbeing.^55^ ^,^ ^56^ A previous study showed that respondents with higher income were happier, more satisfied with their lives, health, achievement, future economic situation, and social conditions.^57^

It is important to highlight that the present findings directly inform the effects of the coronavirus pandemic on the mental health and financial impact in orthodontists during the first stages of the pandemic. At the time when this survey was conducted, Orthodontics and Dentistry in general were lost about how to proceed amidst the chaos of the pandemic. There was no precise scientific evidence regarding the safety of the procedures and care provided. These results may help to draw attention to the need for prevention and control of physiological and financial issues during the coronavirus outbreak, which is still ongoing at the time of this research was conducted. Some changes in the orthodontic practice have come to stay. Garcia-Camba et al.^58^ stated that these changes concern four areas: microbiologic control measures, social distancing measures by redistributing spaces and decreasing the number of patients and companions in the clinics, increasing teleorthodontics and use of appliances and techniques that requires fewer scheduled and urgent appointments, and bioethical considerations to promote a broader view of the psychological aspects of the patients and the community.

A limitation of the present study is the cross-sectional design, since psychological symptoms may change as a consequence of the coronavirus crisis variation. Follow-up studies are needed to complement the present results.

## CONCLUSIONS

In this study performed at the beginning of the pandemic:

» Brazilian orthodontists reported high rates of symptoms of depression, anxiety, insomnia, and distress. Female, graduate students and income below 10k were the most affected.
» Most orthodontists were from moderate to extremely concerned about financial issues and regarding patient care during the pandemic.
» Female orthodontists showed a higher level of financial concern than their male counterparts.
» Professors showed a higher level of financial concern than postgraduate students and clinicians.
» Orthodontists with higher income showed a low level of concern with delay in the orthodontic treatment, contamination after social isolation, and caring of urgencies during the social isolation.
